# Effects of Heat Stress on Metabolite Accumulation and Composition, and Nutritional Properties of Durum Wheat Grain

**DOI:** 10.3390/ijms161226241

**Published:** 2015-12-19

**Authors:** Anna Maria de Leonardis, Mariagiovanna Fragasso, Romina Beleggia, Donatella Bianca Maria Ficco, Pasquale de Vita, Anna Maria Mastrangelo

**Affiliations:** 1 Cereal Research Centre, Council for Agricultural Research and Economics, Foggia 71122, Italy; annamariadeleonardis@libero.it (A.M.D.L.); mariagiovanna.fragasso@gmail.com (M.F.); romina.beleggia@entecra.it (R.B.); donatellabm.ficco@entecra.it (D.B.M.F.); pasquale.devita@entecra.it (P.D.V.); 2 Department of the Sciences of Agriculture, Food and Environment, University of Foggia, S.S. 673 Km 25,200, Foggia 71122, Italy

**Keywords:** durum wheat, heat stress, metabolic profiling, antioxidant activity

## Abstract

Durum wheat (*Triticum turgidum* (L.) subsp. *turgidum* (L.) convar. *durum* (Desf.)) is momentous for human nutrition, and environmental stresses can strongly limit the expression of yield potential and affect the qualitative characteristics of the grain. The aim of this study was to determine how heat stress (five days at 37 °C) applied five days after flowering affects the nutritional composition, antioxidant capacity and metabolic profile of the grain of two durum wheat genotypes: “Primadur”, an elite cultivar with high yellow index, and “T1303”, an anthocyanin-rich purple cultivar. Qualitative traits and metabolite evaluation (by gas chromatography linked to mass spectrometry) were carried out on immature (14 days after flowering) and mature seeds. The effects of heat stress were genotype-dependent. Although some metabolites (e.g., sucrose, glycerol) increased in response to heat stress in both genotypes, clear differences were observed. Following the heat stress, there was a general increase in most of the analyzed metabolites in “Primadur”, with a general decrease in “T1303”. Heat shock applied early during seed development produced changes that were observed in immature seeds and also long-term effects that changed the qualitative and quantitative parameters of the mature grain. Therefore, short heat-stress treatments can affect the nutritional value of grain of different genotypes of durum wheat in different ways.

## 1. Introduction

Durum wheat (*Triticum turgidum* (L.) subsp. *turgidum* (L.) convar. *durum* (Desf.)) is an important crop for human nutrition, especially in the basin of the Mediterranean Sea, where it is mainly used for the production of pasta, traditional/typical bread, couscous, and burghul. Environmental stress can strongly limit the expression of yield potential and affect qualitative characteristics of grain [1,2]. The stress conditions that are commonly experienced by crops are extreme lack or excess of water (*i.e.*, drought, flooding), presence of salt or contaminants (e.g., heavy metals), and temperature changes (*i.e.*, cold, heat). In particular, environmental temperatures have increased since the beginning of the last century and they are predicted to further increase under the present conditions of climate change [3]. This also applies to the major wheat-producing regions [4,5].

The severity of heat stress is a function of the magnitude and rate of temperature increases, as well as the duration of the exposure to a raised temperature [6]. High temperatures can affect grain filling, and during this stage, the optimum temperature for wheat is around 21 °C [7,8]. Moreover, wheat is very sensitive to high temperatures during the reproductive phase, due to direct effects of temperature on grain numbers and dry weight [9,10]. These phenotypic effects are due to molecular modifications at the different levels of gene expression and to changes in metabolite accumulation levels [11].

In durum wheat, the levels of a large number of gene transcripts change in response to heat stress [12]. Moreover, at the post-transcriptional level, heat stress induces significant changes to the durum wheat seed proteome, with effects on polypeptides with allergenic value for sensitive individuals [13,14]. Studies on the responses of wheat to elevated temperatures during the reproductive and grain-filling stages have been carried out [8]. A recent study showed that during wheat-grain filling, high temperatures affect starch biosynthesis, while nitrogen metabolism is favored, thus leading to greater amino acid and protein accumulation [15].

Heat stress can also have strong effects on grain composition in terms of the compounds that are beneficial or detrimental to human health [10,13]. In particular, the accumulation of pigments in the plants, such as carotenoids and anthocyanins, is affected by environmental conditions [16,17,18,19]. Carotenoids are very important elements of the human diet because mammals cannot synthesize vitamin A *de novo*, and β-carotene is a required precursor of vitamin A [20]. Carotenoid pigments show a differential distribution in the wheat seed: α-carotene and β-carotene are prevalently present in the germ, while lutein, which is the most abundant carotenoid, is equally distributed in all of the parts of the seed [21,22,23]. β-Carotene compounds possess antioxidant activity [24], and these antioxidant properties have beneficial effects on human health. Several studies have shown that carotenoids are efficacious in the prevention of age-related macular degeneration and some types of cancer [25,26,27,28]. Anthocyanin pigments have been investigated in many pigmented cereals, such as rice [29,30], maize [31,32], bread and durum wheat [33,34]. These pigments are accumulated in a variety-specific manner in seeds of cereal crops and legumes [32,35,36,37]. They contribute to nonspecific disease resistance in plants [38] and they have a role in the plant response to biotic and abiotic stress [39,40]. They have antioxidant properties [41,42], and they are involved in the plant mechanisms of photoprotection [43,44,45]. In animals, anthocyanins have anti-inflammatory, antimicrobial, and anti-carcinogenic effects [46,47,48]. In humans, anthocyanins have multiple effects on blood vessels that can reduce the risk of coronary heart disease [49,50].

Studies aimed at increasing the total anthocyanin content in wheat grain have been carried out [51]. In the wheat seeds, the anthocyanins are predominant in the external parts [52]. In particular, in purple wheat, the anthocyanin pigments are prevalently located in the pericarp, while in blue wheat they are distributed in the aleurone [53,54]. Greater effects of environmental conditions on anthocyanin accumulation were observed in a spring wheat line with blue aleurone than in wheat genotypes characterized by red and purple seeds [55]. Several tetraploid wheat genotypes with purple grains have been identified, in which there are *Triticum dicoccum* accessions, which originate from Ethiopia. The trait was then introgressed into bread wheat, where this trait has been studied [53,56].

Phenolic compounds represent another class of molecules with beneficial effects on human health, and are widely present in cereal seeds, fruits and vegetables. They have antioxidant activity and are probably involved in the prevention of many chronic human diseases [57,58]. Ferulic acid is abundant among the phenolic compounds and it has antibacterial and antioxidant properties [59,60]. Finally, some studies have shown that free aromatic amino acids [61] and polyamines can also have antioxidant properties [62,63]. Polyamines are aliphatic amines which are present in all living cells [64]. In the body, the pool of polyamines derives from endogenous biosynthesis, intestinal microflora, and the diet [63,64]. Studies on the biological significance of dietary polyamines and on their important roles in supporting the metabolism and maintaining good health have been carried out [65,66,67]. In children, the assumption of polyamines during the first year of life is significantly correlated to prevention of food allergies [68].

Environmental stresses can affect the production and accumulation of these compounds in cereals, thus influencing the antioxidant activity and nutritional quality of grain [17,18,69,70]. In general, stress conditions induce increases in the levels of compounds with antioxidant activity, together with reductions in grain yield [2,8]. It is known that the effects of heat stress on the nutrient status and the photosynthetic performance of a plant, which is important for the mobilization of photoassimilates to the grain, can differently impact the quality of the final product [2,10]; however, there is still poor information on the effects of heat stress on the seed metabolome in relation to its nutraceutical quality.

The present study was aimed at analyzing the effects of heat stress, applied at five days after flowering (DAF), on the metabolic profile and nutritional composition of the grain of two genotypes of durum wheat, one of which is characterized by high levels of anthocyanins in the grain. The data from the present study show that: (i) heat stress applied early during seed development can have strong effects on the mature grain in terms of metabolite accumulation; (ii) there are clear differences in the responses to heat stress of these two durum wheat varieties; and (iii) heat stress influences the antioxidant properties of durum wheat grain in a genotype-dependent manner.

## 2. Results

### 2.1. Effects of Heat Stress on Yield-Related and Qualitative/Nutritional Traits

Two-way ANOVA analysis showed that the effects of Genotype, Treatment and the Genotype × Treatment interactions are highly significant for the protein content of these grains (Table 1). However, only the effects of Genotype were significant for carotenoid content, while for antioxidant activity, the effects of Treatment and the Genotype × Treatment interaction were highly significant (Table 1).

**Table 1:** ANOVA analysis of the yield-related and qualitative/nutritional traits.

Seeds	Trait	G	T	G × T	“Primadur”		“T1303”	
					Control	Heat Shocked	Control	Heat Shocked
Mature	Protein content (%)	****	***	**	14.94 ± 0.52 ^c^	15.04 ± 0.25 ^c^	16.74 ± 0.42 ^b^	19.00 ± 0.33 ^a^
	Carotenoids (µg/g·dw)	***	ns	ns	9.26 ± 0.54 ^a^	8.67± 0.23 ^a^	3.5 ± 0.49 ^b^	3.56 ± 0.04 ^b^
	Anthocyanins (µg/g·dw)	-	-	-	nd	nd	9.3 ± 0.54 ^b^	16 ± 0.73 ^a^
	Antioxidant activity (mM Trolox/kg·dw)	ns	***	***	13.64 ± 0.02 ^b^	13.85 ± 0.13 ^b^	12.95 ± 0.13 ^b^	14.2 ± 0.28 ^a^
	Individual grain weight (mg·dw)	****	****	ns	28.2 ± 0.1 ^c^	25.4 ± 1 ^d^	52.2 ± 0.32 ^a^	47.8 ± 1.3 ^b^
	Grain yield per spike (mg·dw)	***	***	ns	1410 ± 38 ^a^	1184 ± 89 ^b^	1210 ± 13 ^b^	1093 ± 5.8 ^b^
	Grain number per spike	****	ns	ns	50 ± 2 ^a^	47 ± 5 ^a^	23 ± 1 ^b^	23 ± 1 ^b^
Immature	Individual grain weight (mg·dw)	****	ns	ns	10.8 ± 0.97 ^b^	11.2 ± 0.81 ^b^	15.9 ± 0.41 ^a^	16.3 ± 0.24 ^a^
	Grain yield per spike (mg·dw)	****	ns	ns	513 ± 20.1 ^a^	536 ± 22.3 ^a^	354 ± 6.5 ^b^	370 ± 19.1 ^b^
	Grain number per spike	****	ns	ns	49 ± 2 ^a^	48 ± 4 ^a^	23 ± 1 ^b^	22 ± 2 ^b^

ns, not significant; **, *p* < 0.01; ***, *p* < 0.001; ****, *p* < 0.0001; nd, not detected; -, not calculated; Values in the same row followed by different superscript letters are significantly different (*p* < 0.05); dw, dry weight; G, Genotype; T, Treatment; G × T, Genotype × Treatment interaction.

Higher protein content was seen for “T1303” compared to “Primadur” in both the control and the heat-stressed plants, and the protein content increased significantly in response to the heat stress only for “T1303”. “Primadur” showed higher carotenoid content with respect to “T1303” (*i.e.*, about three-fold greater), although no significant variations in carotenoids were observed in response to the heat stress for either of these genotypes. Anthocyanins were measured as cyanidin-3-glucoside equivalents, and these were detected only for “T1303”, as expected, with a significant increase in response to the heat stress (Table 1). The antioxidant activity showed very similar levels for the “Primadur” control and heat-stressed conditions, and for the “T1303” heat-stressed wholemeal samples. These values were significantly higher with respect to those observed for the “T1303” control, and therefore, for the “T1303” alone, the heat stress induced an increase in antioxidant activity.

For the yield-related parameters, the effects of both Genotype and Treatment were highly significant for the individual grain weight and grain yield per spike for the mature grains. However, only the effect of Genotype was highly significant for these two traits for the immature grain.

Heat stress resulted in significant changes for the individual grain weight and grain yield per spike for the mature seeds of both of these durum wheat genotypes, but not for the immature seeds. Statistically significant weight losses of 11.0% (from 28.2 ± 0.1 to 25.4 ± 1.05 mg) and 8.4% (from 52.2 ± 0.32 to 47.8 ± 1.3 mg) were observed for “Primadur” and “T1303”, respectively, in response to the heat stress. However, the grain yield per spike was not statistically different across the “T1303” control and the heat-stressed “Primadur” and “T1303”.

### 2.2. Analysis of Variance for Classes of Polar Metabolites

Metabolic profiling of the whole-grain samples led to the identification of 42 metabolites that were included in five classes of compounds: amino acids, *N*-compounds, organic acids, sugars, and sugar alcohols. ANOVA analysis assuming a random model was carried out for the different classes of compounds to determine the contribution of each variance component, as Genotype, Treatment, and timing (immature and mature seeds), and their interactions (Table 2).

**Table 2:** ANOVA analysis for each class of polar metabolite.

Class of Metabolite	G	T	t	G × T	G × t	T × t	G × T × t
Amino acids	***	***	***	***	***	***	***
*N*-compounds	***	**	***	***	***	ns	ns
Organic acids	***	**	***	***	***	*	***
Sugars	***	*	***	***	***	ns	**
Sugar alcohols	***	*	***	***	***	ns	**

ns, not significant; *, *p* < 0.05; **, *p* < 0.01; ***, *p* < 0.001; G, Genotype; T, Treatment; t, timing; and their interactions.

The effects of Genotype, timing and the Genotype × Treatment and Genotype × timing interactions were highly significant for all classes of compound, and for the total amino acids the effect of Treatment was also highly significant.

**Figure 1:**
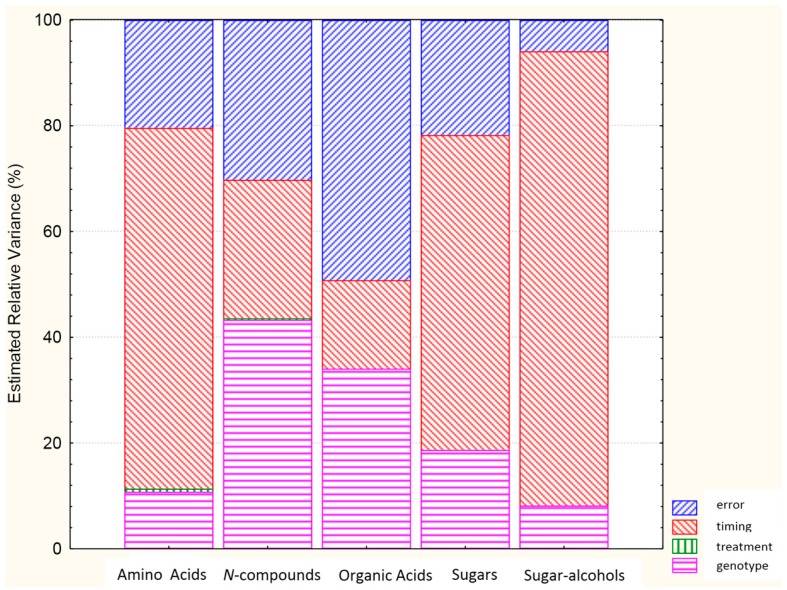
Analysis of the relative variance for each class of metabolite.

The levels of each class of metabolite were higher for “Primadur” than “T1303” (Table S1), and the immature seeds showed higher levels of these metabolites with respect to the mature seeds (Table S1). All of the classes of compounds analyzed showed general increases in response to the heat stress (Table S1). Analysis of the relative variance showed that the largest part of the variance was represented by timing for the amino acids, sugar, and sugar alcohols, whereas Genotype represented the largest part of the variance for the *N*-compounds and organic acids (Figure 1). These data suggested the need to perform the statistical analysis for the individual metabolites separately for the immature and mature seeds, and to evaluate the individual responses of the two genotypes to heat stress.

### 2.3. Analysis of Variance for Individual Metabolites

Sixty-two percent and 36% percent of the compounds evaluated here showed statistically significant differences between the control and heat-stress conditions for the immature and mature seeds, respectively. The Genotype effect was significant for all of the free amino acids assessed for the immature seeds, with the exception of leucine; on the contrary, only the levels of valine, isoleucine, and asparagine were significantly different between the two genotypes for the mature seeds (Table 3). The effects of Treatment were significant for the free amino acids except valine, glutamic acid, serine, threonine, leucine and β-alanine for the immature seeds, whereas Treatment was significant only for valine and glycine for the mature seeds, in which a very strong effect of the interaction was seen. The effects of Genotype on the levels of *N*-compounds were highly significant for the immature seeds, while the effects of the Genotype × Treatment interaction were highly significant for the mature seeds. For putrescine and cadaverine, the effects of Genotype, Treatment, and the Genotype × Treatment interaction were significant for both the immature and mature seeds, whereas for spermidine only the effects of Genotype and the Genotype × Treatment interaction were highly significant for the immature and mature seeds, respectively. The highest levels of significance were for Genotype and the Genotype × Treatment interaction for organic acids for the immature seeds. For ferulic acid, the effects of Genotype, Treatment and the Genotype × Treatment interaction were highly significant.

**Table 3:** ANOVA analysis performed separately for the immature and mature seeds.

Metabolite	ANOVA Significance					
	Immature Seeds			Mature Seeds		
	G	T	G × T	G	T	G × T
Amino acids	****	***	***	ns	ns	****
Valine	****	ns	ns	*	****	****
Alanine	****	****	*	ns	ns	****
Asparagine	****	**	***	**	ns	****
Aspartic acids	****	***	****	ns	ns	****
Glutamic acid	****	ns	ns	ns	ns	****
Isoleucine	***	**	ns	**	ns	****
Serine	**	ns	ns	ns	ns	****
Glycine	****	**	**	ns	**	****
Threonine	****	ns	ns	ns	ns	****
Leucine	ns	ns	ns	ns	ns	*
β-Alanine	****	ns	ns	ns	ns	*
Phenylalanine	****	****	****	ns	ns	****
Tryptophan	****	**	ns	ns	ns	***
Tyrosine	****	**	*	ns	ns	****
GABA	****	**	**	ns	ns	****
*N*-compounds	***	*	*	*	**	****
Putrescine	****	**	**	*	*	*
Cadaverine	****	***	***	*	***	**
Spermidine	****	ns	ns	ns	ns	****
Organic acids	****	**	***	ns	ns	****
Citric acid	****	ns	***	ns	ns	****
Ferulic acid	****	****	****	*	*	****
Fumaric acid	****	**	****	**	*	****
Malic acid	****	*	****	**	**	****
Nicotinic acid	****	ns	ns	ns	ns	***
Oxalic acid	****	ns	****	ns	ns	***
Quinic acid	****	ns	ns	ns	ns	****
Saccharic acid	****	*	*	*	**	****
Shikimic acid	****	ns	ns	*	ns	ns
3PGA	****	****	****	****	****	****
Sugars	****	ns	***	*	*	****
Raffinose	****	ns	**	ns	ns	****
Sucrose	ns	***	**	ns	ns	****
Glucose	****	****	****	**	**	****
Glucose 6-phosphate	****	ns	ns	*	*	**
Fructose	****	***	****	ns	ns	****
Fructose 6-phosphate	****	ns	ns	ns	ns	***
Ribose	****	***	***	ns	ns	****
Mannose	****	**	**	ns	ns	****
Palatinose and maltitol	****	****	****	****	****	****
Xylose and lyxose	****	****	****	ns	ns	****
Maltose and turanose	****	***	***	**	*	****
Sugar alcohols	****	ns	ns	ns	**	****
Mannitol	****	*	**	**	****	****
*myo*-Inositol	****	*	**	ns	ns	****
Glycerol	****	ns	ns	***	****	****

ns, not significant; *, *p* < 0.05; **, *p* < 0.01; ***, *p* < 0.001; ****, *p* < 0.0001; GABA, γ-4-aminobutyric acids; 3PGA, 3-phosphoglyceric acid; G, Genotype; T, Treatment; G × T, Genotype × Treatment interaction.

The effects of the Genotype × Treatment interaction were highly significant for all of the organic acids for the mature seeds, with the exception of shikimic acid, for which only the effect of Genotype was significant (Table 3; *p* < 0.05). Treatment affected the accumulation of some of the organic acids (*i.e.*, malic, fumaric, ferulic, 3PGA, saccharic) for both the immature and mature seeds. For the immature seeds, the effects of Genotype were highly significant for the sugars, with the exception of sucrose, whereas the effects of Treatment and the Genotype × Treatment interaction were not significant for any of the sugars. On the contrary, for the mature seeds, the effects of the Genotype × Treatment interaction were highly significant for the sugars, whereas the effects of Genotype and Treatment were significant for glucose, glucose 6-phosphate, palatinose, maltitol, maltose, and turanose. The effects of Genotype on the variability of all of the sugar alcohols were highly significant for the immature seeds, whereas Treatment and the Genotype × Treatment interaction had significant effects on the variability of the sugar alcohols for the mature seeds.

### 2.4. Metabolite Changes in Response to Heat Stress in the Two Durum Wheat Genotypes

Ninety-five percent and 38% of these compounds evaluated here showed statistically significant differences between the two genotypes for the immature and mature seeds, respectively (Table 3). Following the heat stress, all of the classes of compounds analyzed, except for the *N*-compounds, showed general increases in their levels for “Primadur” and general decreases in “T1303” (Table S2). The analytical data for each metabolite, the analysis of variance, and the least significant difference test among the means of all of the detected compounds are reported in Table 4. In general, the most abundant metabolites were sugars, and among these, raffinose had the highest levels, followed by sucrose.

**Figure 2:**
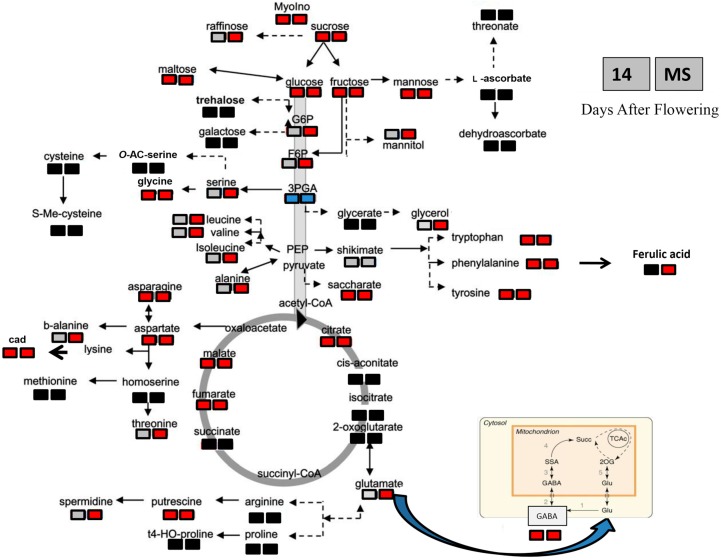
Changes in the metabolite profiles for the “Primadur” seeds, for the control compared to the heat-stressed conditions, for the immature (14 DAF) and mature (MS) stages of seed development. Changes in the metabolite levels were calculated as the ratios between the levels for the heat stress and the control. Anthocyanins were not detected. To visualize the changes, significant increases and decreases are indicated in red and blue, respectively, within a metabolic scheme (*p* < 0.05; Student’s *t* tests). b-alanine, β-Alanine; GABA, γ-4-aminobutyric acids. Solid line arrows: single step reactions; dash line arrows: pathways composed of more than one reaction; light gray shape: compounds which are not significantly different in seeds from heat stressed plants and control; red shape: compounds whose level is higher in seeds from heat stressed plants compared to control; blue shape: compounds whose level is lower in seeds from heat stressed plants compared to control; black shape: compounds not evaluated in the present study. The metabolic scheme is modified from [71].

**Figure 3:**
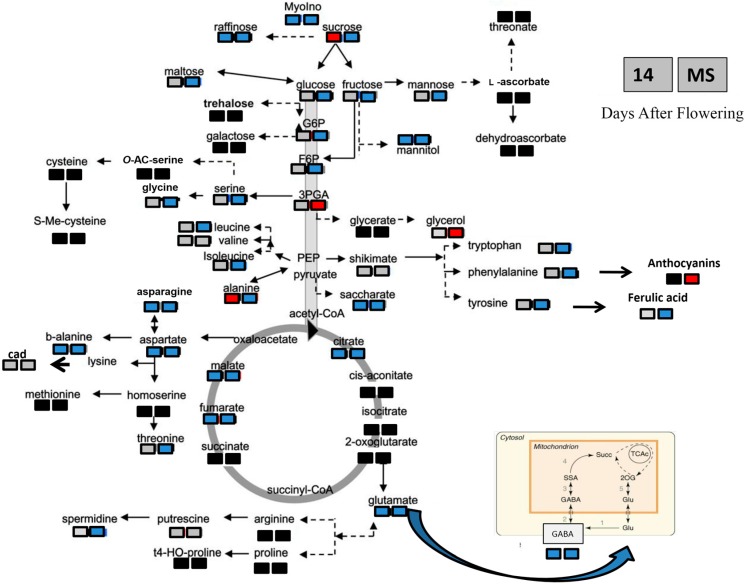
Changes in metabolite profiles for the “T1303” seeds, for the control compared with the heat-stressed conditions, for the immature (14 DAF) and mature (MS) stages of seed development. Changes in the metabolite levels were calculated as the ratios between the levels for the heat stress and the control. To visualize the changes, significant increases and decreases are indicated in red and blue, respectively, within a metabolic scheme (*p* < 0.05; Student’s *t* tests). b-alanine, β-Alanine; GABA, γ-4-aminobutyric acids. Solid line arrows: single step reactions; dash line arrows: pathways composed of more than one reaction; light gray shape: compounds which are not significantly different in seeds from heat stressed plants and control; red shape: compounds whose level is higher in seeds from heat stressed plants compared to control; blue shape: compounds whose level is lower in seeds from heat stressed plants compared to control; black shape: compounds not evaluated in the present study. The metabolic scheme is modified from [71].

Based on the large differences between the two genotypes, we decided to use Student’s test to evaluate the significance of variation in response to heat stress for each individual metabolite at both stages of seed development (Table S3). Although very different behaviors were seen for the two genotypes, some of the metabolites (*i.e.*, sucrose, glycerol, alanine) increased in response to the heat stress for both “Primadur” and “T1303” (Figure 2 and Figure 3). Differences in the timing of the responses to the heat stress for each individual metabolite were observed. In “Primadur”, some of the amino acids (*i.e.*, asparagine, aspartic acid, γ-4-aminobutyrric acid (GABA), phenylalanine, tyrosine, glycine, tryptophan) increased in response to heat stress for both stages of seed development, whereas other amino acids increased only for the mature seeds. For the immature seeds of “T1303”, alanine increased in response to the heat stress, whereas the other amino acids did not change or decreased (*i.e.*, glutamic acid, β-Alanine, asparagine, aspartic acid, GABA); for the mature seeds, all of the amino acids decreased, with the exception of valine, which did not change. For the *N*-compounds, the levels of putrescine and cadaverine increased for “Primadur”, whereas these did not change for “T1303”, while spermidine increased and decreased for the mature seeds of “Primadur” and “T1303”, respectively. Among the organic acids, the levels of shikimic acid did not change in either durum wheat genotype, while the other organic acids increased and decreased for the mature seeds of “Primadur” and “T1303”, respectively. Among the sugars and sugar alcohols, the levels of sucrose for the immature seeds and glycerol for the mature seeds increased in response to the heat stress for both of these genotypes.

**Table 4:** Metabolite composition of the seeds.

Metabolite Class	Metabolite	Metabolite Content According to Seeds, Genotype and Heat Stress (µg/g DW)							
		Immature Seeds				Mature Seeds			
		“Primadur”		“T1303”		“Primadur”		‘T1303’	
		Control	Heat Shocked	Control	Heat Shocked	Control	Heat Shocked	Control	Heat Shocked
Amino acids	Valine	270.70 ± 21.41 ^b^	267.00 ± 7.2 ^b^	472.61 ± 33.09 ^a^	417.28 ± 31.48 ^a^	4.61 ± 0.36 ^c^	35.71 ± 1.9 ^a^	18.64 ± 0.78 ^b^	12.41 ± 4.65 ^b^
	Alanine	564.62 ± 141.78 ^c^	872.74 ± 163.77 ^b^	1101.31 ± 64.53 ^b^	1755.42 ± 33.55 ^a^	2.30 ± 1.51 ^b^	52.33 ± 10.07 ^a^	46.00 ± 13.9 ^a^	2.54 ± 0.64 ^a^
	Asparagine	1288.58 ± 240.59 ^b^	2937.24 ± 463.81 ^a^	762.11 ± 108.55 ^c^	399.26 ± 28.08 ^c^	94.80 ± 22.3 ^d^	785.64 ± 153.1 ^b^	1130.97 ± 198.7 ^a^	251.81 ± 17.4 ^c^
	Aspartic acid	905.30 ± 99.02 ^b^	1594.59 ± 91.14 ^a^	234.81 ± 36.14 ^c^	109.90 ± 13.69 ^c^	12.18 ± 6.4 ^b^	731.34 ± 128.6 ^a^	651.2 ± 134.8 ^a^	19.89 ± 2 ^b^
	Glutamic acid	1783.16 ± 38.36 ^b^	2044.83 ± 282.1 ^a^	225.47 ± 12.81 ^c^	170.25 ± 11.73 ^c^	48.8 ± 10.14 ^b^	922.88 ± 188.47 ^a^	843.14 ± 71.3 ^a^	58.70 ± 4.02 ^b^
	Isoleucine	102.73 ± 5.5 ^a^	79.90 ± 12.47 ^b^	145.72 ± 14.61 ^a^	122.15 ± 9.02 ^a^	1.82 ± 1.36 ^b^	7.56 ± 1.25 ^a^	8.91 ± 0.85 ^a^	5.53 ± 0.55 ^a^
	Serine	1288.70 ± 275.98 ^a^	1239.40 ± 200.18 ^a^	816.27 ± 40.28 ^b^	871.37 ± 50.38 ^b^	11.49 ± 0.37 ^b^	507.24 ± 111.58 ^a^	452.58 ± 102.7 ^a^	43.95 ± 35.5 ^b^
	Glycine	388.48 ± 30.45 ^b^	477.34 ± 4.29 ^a^	364.77 ± 15.54 ^c^	349.93± 12.33 ^c^	0.93 ± 0.04 ^c^	11.46 ± 1.9 ^a^	7.08 ± 1.45 ^b^	2.22 ± 1.53 ^c^
	Threonine	377.24 ± 26.29 ^a^	370.0 ± 18.08 ^a^	114.34 ± 12.16 ^b^	111.11 ± 4.75 ^b^	24.90 ± 1.46 ^b^	188.53 ± 36.85 ^a^	193.26 ± 9.17 ^a^	24.96 ± 18.12 ^b^
	Leucine	54.16 ± 12.83	44.22 ± 10.61	66.34 ± 9.04	57.12 ± 8.96	1.76 ± 0.51	3.87 ± 1.54	4.11 ± 0.8	2.73 ± 0.56
	β-Alanine	36.84 ± 0.58 ^a^	41.59 ± 4.86 ^a^	3.25 ± 0.3 ^b^	2.31 ± 0.06 ^b^	1.29 ± 1.8 ^b^	24.36 ± 4.9 ^a^	20.39 ± 1.8 ^a^	0.15 ± 0.04 ^b^
	Phenyl-alanine	122.70 ± 5.13 ^b^	184.59± 2.87 ^a^	17.56 ± 1.47 ^c^	15.45 ± 0.53 ^c^	27.73 ± 0.4 ^b^	74.41 ± 10.9 ^a^	75.69 ± 9 ^a^	33.67 ± 0.4 ^b^
	Tryptophan	374.54 ± 20.03 ^c^	495.99 ± 76.84 ^c^	885.13 ± 119.2 ^b^	1174.36 ± 76.73 ^a^	217.38 ± 15.8 ^b^	328.26 ± 14.5 ^a^	288.04 ± 51.8 ^a^	177.44 ± 32.7 ^c^
	Tyrosine	42.84 ± 2.79 ^b^	59.27 ± 7.69 ^a^	7.09 ± 1.32 ^c^	8.64 ± 1.16 ^c^	2.27 ± 1.2 ^b^	23.78 ± 7.4 ^a^	25.42 ± 4.8 ^a^	4.30 ± 2.8 ^b^
	GABA	787.32 ± 48.17 ^b^	1101.48 ± 114.79 ^a^	37.75 ± 6.45 ^c^	14.06 ± 4.3 ^c^	8.44 ± 0.99 ^b^	403.25 ± 102.8 ^a^	335.83 ± 37.7 ^a^	7.54 ± 0.84 ^b^
*N*-compounds	Putrescine	22.93 ± 3.8 ^b^	39.68 ± 7.3 ^a^	2.35 ± 0.2 ^c^	2.46 ± 0.4 ^c^	0.21 ± 0.05 ^b^	11.22 ± 6.7 ^a^	0.72 ± 0.56 ^b^	0.73 ± 0.3 ^b^
	Cadaverine	7.13 ± 0.8 ^b^	10.49 ± 0.7 ^a^	0.52 ± 0.07 ^c^	0.43 ± 0.03 ^c^	0.52 ± 0.07 ^b^	3.48 ± 0.6 ^a^	0.82 ± 0.7 ^b^	1.59 ± 0.06 ^b^
	Spermidine	38.22 ± 1.7 ^a^	47.45 ± 16.4 ^a^	1.34 ± 0.2 ^b^	1.88 ± 1 ^b^	0.21 ± 0.05 ^b^	10.68 ± 5.1 ^a^	14.57 ± 2.8 ^a^	0.21 ± 0.05 ^b^
Organic acids	Citric acid	459.28 ± 64.1 ^b^	634.92 ± 41.6 ^a^	177.07 ± 12.5 ^c^	81.50 ± 16.1 ^d^	1.74 ± 0.18 ^b^	287.19 ± 94.4 ^a^	274.99 ± 28 ^a^	4.99 ± 0.42 ^b^
	Ferulic acid	10.32 ± 0.7 ^b^	19.34 ± 0.3 ^a^	0.71 ± 0.08 ^c^	0.50 ± 0.15 ^c^	0.24 ± 0.3 ^c^	8.70 ± 1.5 ^a^	5.85 ± 1.1 ^b^	0.08 ± 0.01 ^c^
	Fumaric acid	177.35 ± 2.9 ^b^	217.43 ± 7.4 ^a^	97.94 ± 11.2 ^c^	18.34 ± 1.4 ^d^	27.47 ± 0.2 ^c^	153 ± 16 ^a^	113.33 ± 5.1 ^b^	23.34 ± 10.8 ^c^
	Malic acid	3644.35 ± 217.6 ^b^	4643.75 ± 122.3 ^a^	1423.95 ± 82.4 ^c^	998.79 ± 156.1 ^d^	388.73 ± 11.8 ^c^	1714.39 ± 371 ^b^	2485.56 ± 113.5 ^a^	384.11 ± 13.7 ^c^
	Nicotinic acid	12.5 ± 1.6 ^a^	13.61 ± 0.2 ^a^	7.70 ± 0.3 ^b^	7.05 ± 0.7 ^b^	3.70 ± 1.1 ^c^	5.97 ± 0.22 ^b,c^	7.23 ± 1.5 ^a,b^	3.50 ± 0.8 ^c,d^
	Oxalic acid	195.75 ± 4.2 ^c^	278.72 ± 15.9 ^a^	227.04 ± 6.7 ^b^	133.30 ± 10.7 ^d^	190.53 ± 58.6 ^b^	403.1 ± 44.6 ^a^	355.3 ± 56.7 ^a,c^	172.87 ± 60.9 ^b,c^
	Quinic acid	159.48 ± 52.6 ^a^	160.95 ± 20 ^a^	4.63 ± 0.24 ^b^	3.65 ± 0.05 ^b^	1.1 ± 0.61 ^b^	33.52 ± 14.1 ^a^	40.93 ± 10.1 ^a^	0.24 ± 0.01 ^b^
	Saccharic acid	6478.77 ± 828.1 ^b^	9090.44 ± 1296.2 ^a^	1573.27 ± 22.5 ^c^	1299.86 ± 71.3 ^c^	108.98 ± 8.9 ^c^	5359.32 ± 277.3 ^a^	4525.53 ± 335.5 ^b^	188.93 ± 5.7 ^c^
	Shikimic acid	22.21 ± 4.4 ^a^	26.66 ± 10.4 ^a^	1.46 ± 0.3 ^b^	0.97 ± 0.1 ^b^	8.86 ± 0.26	11.59 ± 2.7	15.36 ± 4.3	14.40 ± 1.8
	3PGA	5.67 ± 0.2 ^a^	3.7 ± 0.1 ^b^	0.38 ± 0.07 ^c^	0.38 ± 0.04 ^c^	26.19 ± 0.7 ^b^	4.17 ± 0.6 ^c^	4.49 ± 1.5 ^c^	35.63 ± 0.8 ^a^
Sugars	Raffinose	171 × 10^3^ ± 23,837 ^a^	194.2 × 10^3^ ± 17,216 ^a^	109.9 × 10^3^ ± 12,833 ^b^	51.1 × 10^3^ ± 3581 ^c^	23.6 × 10^3^ ± 488 ^b^	79.3 × 10^3^ ± 17,194 ^a^	60 × 10^3^ ± 11,498 ^a^	29.1 × 10^3^ ± 1801 ^b^
	Sucrose	74.1 × 10^3^ ± 4287 ^b^	93.6 × 10^3^ ± 6549 ^a^	79.9 × 10^3^ ± 1056 ^b^	83.7 × 10^3^ ± 958 ^a^^,b^	22.8 × 10^3^ ± 367 ^b^	54.7 × 10^3^ ± 7948 ^a^	50.5 × 10^3^ ± 4090 ^a^	23.4 × 10^3^ ± 546 ^b^
	Glucose	6333.51 ± 183 ^b^	8777.81 ± 208 ^a^	609.21 ± 38 ^c^	453.68 ± 123 ^c^	5.26 ± 0.4 ^c^	5626.15 ± 517 ^a^	3179.70 ± 763 ^b^	8.00 ± 0.5 ^c^
	Glucose 6-phosphate	48.37 ± 9.3 ^a^	52.1 ± 11.1 ^a^	0.32 ± 0.04 ^b^	0.18 ± 0.07 ^b^	0.18 ± 0.07 ^c^	41.67 ± 12 ^b^	141.7 ± 71 ^a^	0.32 ± 0.03 ^c^
	Fructose	15,186.55 ± 1824 ^b^	22,969.97 ± 188 ^a^	1178,03 ± 395 ^c^	808.44 ± 100 ^c^	502.52 ± 376 ^b^	13,476.94 ± 2103 ^a^	8658.34 ± 3269 ^a^	284.88 ± 54 ^b^
	Fructose 6-phosphate	919.3 ± 139 ^a^	736.7 ± 307 ^a^	3.8 ± 0.8 ^b^	2.63 ± 0.5 ^b^	0.86 ± 0.56 ^b^	345.09 ± 135 ^a^	316.75 ± 105 ^a^	2.8 ± 0.71 ^b^
	Ribose	257.68 ± 28.6 ^b^	367.70 ± 17.2 ^a^	15.72 ± 1.2 ^c^	17.09 ± 0.4 ^c^	4.33 ± 0.2 ^b^	127.98 ± 15 ^a^	140.92 ± 32.5 ^a^	2.48 ± 0.08 ^b^
	Mannose	1749.64 ± 223 ^b^	2780.72 ± 294 ^a^	124.04 ± 37 ^c^	117.94 ± 11 ^c^	27.44 ± 1.3 ^b^	1596.85 ± 34.3 ^a^	1461.67 ± 284 ^a^	31.77 ± 8.8 ^b^
	Palatinose and maltitol	23.19 ± 2.5 ^b^	74.29 ± 8.7 ^a^	9.53 ± 2.1 ^c^	15.14 ± 1.9 ^c^	20.30 ± 9.5 ^c^	22.91 ± 9.5 ^b,c^	134.31 ± 17 ^a,b^	21.15 ± 5.4 ^c,d^
	Xylose and lyxose	236.62 ± 17.8 ^b^	914.38 ± 51 ^a^	42.55 ± 2.8 ^c^	35.72 ± 3 ^c^	6.80 ± 0.6 ^c^	229.21 ± 38.6 ^a^	155.01 ± 37.5 ^b^	17.26 ± 2.4 ^c^
	Maltose and turanose	32.6 × 10^3^ ± 2528 ^b^	50 × 10^3^ ± 4071 ^a^	117.6 × 10^3^ ± 348 ^c^	20.1 × 10^3^ ± 3258 ^c^	7176.69 ± 132 ^c^	30.1 × 10^3^ ± 4058 ^a^	21.3 × 10^3^ ± 1875 ^b^	4382.71 ± 399 ^d^
Sugar alcohols	Mannitol	133.43 ± 12.33 ^a^	140.58 ± 6 ^a^	41.39 ± 3.7 ^b^	11.96 ± 1.1 ^c^	2.90 ± 0.5 ^d^	87.08 ± 5.5 ^a^	62.05 ± 3.3 ^b^	10.37 ± 2.1 ^c^
	*myo*-Inositol	335.32 ± 12.9 ^b^	364.13 ± 6.5 ^a^	61.86 ± 3 ^c^	52.99 ± 1.3 ^c^	10.96 ± 0.88 ^b^	256.76 ± 48.8 ^a^	227.33 ± 43.1 ^a^	14.98 ± 0.45 ^b^
	Glycerol	1698.32 ± 73 ^a^	1733.06 ± 128 ^a^	1181.24 ± 60 ^b^	1229.17 ± 79 ^b^	16.06 ± 0.96 ^c^	128.94 ± 14.9 ^a^	40.61 ± 4.6 ^b^	49.77 ± 2.9 ^b^

Data are means ± standard deviation. Values in the same row followed by different superscript letters are significantly different (*p* < 0.05; Tukey’s tests). No letters are given when differences are not statistically different. ANOVA analysis was performed independently on immature and mature seeds. GABA, γ-4-aminobutyric acids; 3PGA, 3-phosphoglyceric acid.

## 3. Discussion

### 3.1. Heat Shock Applied Early during Seed Development Produces Different Long-Term Effects in Two Durum Wheat Genotypes

A significant reduction in the grain weight was induced by the heat stress for both durum wheat genotypes used in the present study. The decrease in the grain weight was greater for “Primadur” than for “T1303”, while the grain numbers per spike did not change significantly in response to the heat stress for either of the genotypes. These data indicate that heat shock was effective in producing a clear effect on the developing seeds, and they suggest that “Primadur” was more susceptible to the heat stress than “T1303”. The effects of heat stress were evident not only in terms of the grain weight, but also in terms of the protein content and metabolite accumulation. The protein content increased in response to the heat stress only for “T1303”. For metabolite accumulation, 62% and 36% of the compounds evaluated here showed statistically significant differences between the control and stress conditions for the immature and mature seeds, respectively. Similar findings were reported for mature maize grains, in which from 17% to 37% of the compounds included showed statistically significant differences that depended on the growing season [72]. These data underline that the effects of heat stress are stronger for the immature seeds, but, interestingly, most of the changes observed a few days after the stress imposition were maintained up to grain maturity.

Indeed, three types of behaviors in response to the heat stress were observed: for the total amino acids and organic acids, the effects of the heat treatment were significant only for the immature seeds; for the sugar and sugar alcohols, the effects of the heat treatment were significant only for the mature seeds; for the *N*-compounds, however, the effects of the heat treatment were significant for both the immature and mature seeds. The strong effects of the heat treatment at both stages of seed development for the polyamines (*i.e.*, *N*-compounds) can be explained according to their roles in plant hormone signal transduction pathways in response to abiotic stress [73,74,75,76]. There is evidence that putrescine and abscissic acid are part of a positive feedback loop, in which each one induces the biosynthesis of the other following abiotic stress treatments [70,77]. Abscissic acid also enhanced the increase of polyamine (*i.e.*, putrescine, spermidine, spermime) amounts, and induced the polyamine oxidation pathway in grapevine in response to drought stress, which resulted in secondary protective effects, such as stomata closure [78]. When we consider the effects of Genotype and the Genotype × Treatment interaction, the first was highly significant for the immature seeds (95% of the analyzed compounds), and the second was highly significant for the mature seeds (98% of the analyzed compounds). These data are compatible with different responses to stress of these two genotypes; “Primadur” and “T1303” showed general increases and decreases, respectively, for all of the classes of polar metabolites in response to the heat stress.

Moreover, the content of 62% of analyzed metabolites increased for the immature seeds of “Primadur”, and the same response to stress was observed for 95% of the metabolites analyzed for the mature seeds. The content of eight amino acids and polyamines increased for the immature seeds, with the same behavior maintained for the mature seeds. For the sugars, the levels of raffinose, glucose 6-phosphate and fructose 6-phosphate did not change in response to the heat stress for the immature seeds and increased for the mature seeds, whereas palatinose and maltitol increased only for the immature seeds. In response to the heat stress, for “T1303”, the content of 40% of the analyzed metabolites decreased for the immature seeds, and the same response was observed for the mature seeds, while the content of 55% of the analyzed metabolites, including ferulic acid and the polyamines, did not change for the immature seeds and decreased for the mature seeds. Here also alanine, palatinose and maltitol increased for the immature seeds and decreased for the mature seeds. These different behaviors for these responses to heat stress in the “T1303” genotype with respect to the “Primadur” genotype might be due to differences in the timing of the metabolite induction between them, such that for “T1303”, some metabolites might be induced by the heat stress at a stage not considered in this analysis, or these genotypes might show differences in stress perception and tolerance. As an example, increases in the cadaverine and putrescine content were observed in response to heat stress only for “Primadur”, which would suggest a higher sensitivity with respect to “T1303”, according to the higher weight reduction of the grain observed in “Primadur” compared to “T1303” and to previous studies that have shown stress-induced selective accumulation of polyamines in sensitive plant species and wheat varieties [79,80,81]. Similarly, the level of GABA increased in response to the heat stress only for “Primadur”. Increases in GABA levels after heat stress are well documented in the literature as, following high temperatures, the increase in the cytosolic levels of Ca^2+^ can lead to calmodulin-mediated activation of glutamate decarboxylase, the enzyme that catalyzes the synthesis of GABA from glutamate [82,83]. Furthermore, under oxidative stress conditions, degradation of GABA can also limit the accumulation of reactive oxygen intermediates [84], and therefore, the differences observed in the present study might be related to different abilities to reduce the accumulation of reactive oxygen species in “T1303” with respect to “Primadur”. Finally, some of the differences observed in the metabolite levels for the “Primadur” and “T1303” grain can be related to the accumulation of anthocyanins in “T1303”. Sugar metabolism can be affected by the synthesis of anthocyanins, which are present in plants in their glycosylated forms, usually bound to sugars as glucose, galactose, arabinose, rhamnose, xylose, and fructose [85,86,87]. For the mature seeds derived from “T1303”, when heat-stressed, with respect to the control plants, this might explain the decrease of many sugars and some related compounds, such as *myo*-inositol, which regulates the production of galactinol, a precursor of raffinose [88,89]. Sugars, such as the raffinose family, oligosaccharides, and sugar alcohols, have antioxidant properties and protect plant cells from oxidative stress through the maintenance of the redox homeostasis [90].

Furthermore, if we consider the control conditions, for the mature seeds, “T1303” was characterized by high levels of sugars compared to “Primadur”, and this confirmed previous studies carried out in other species. A higher concentration of glucose, galactose and *myo*-inositol was observed in the flesh of the mature fruit of the red “Anjou” pear, with respect to the green fruit [91]. Moreover, red and purple genotypes of potato can accumulate up to 30% and 60% higher sucrose and glucose levels with respect to the white and yellow genotypes [92]. Also, in black rice, the contents of sugars, sugar alcohols and proteins were higher with respect to non-colored rice [93,94]. Similar results were found for raffinose in pears [91].

Despite the large differences in the metabolite changes in response to heat stress between these two genotypes, where 95% of the metabolites increased for the “Primadur” mature seeds in contrast to 81% of the metabolites that decreased for the “T1303” mature seeds (except for 3PGA and glycerol, which increased), it is interesting to observe that some metabolites showed the same behavior for both genotypes. These included some amino acids (*i.e.*, threonine, serine, leucine, valine), spermidine and nicotinic acid, which did not change for immature seeds, shikimic acid, which did not change for both immature and mature seeds, and glycerol and 3PGA, which did not change for immature seeds and increased for mature seeds. Some of the responses in terms of the metabolite amounts were clearly related to the heat-stress response, and both genotypes were indeed characterized by an increase in sucrose for immature seeds and in glycerol for mature seeds from heat-stressed plants, with respect to the controls. Sucrose can act as a signaling molecule, and in *Arabidopsis* leaves, it was shown that sucrose increased very rapidly in response to temperature shock to maintain high levels for the duration of the stress treatment [95]. While most studies describe the effect of heat stress on vegetative tissues of plants [96], in the present study, we show that heat-induced accumulation of sucrose and glycerol is part of the mechanism of response to heat stress for seeds also. In agreement with these data, heat stress applied a few days after flowering induced the accumulation of sucrose in the developing caryopsis of rice [83].

### 3.2. Effects of Heat Stress on Antioxidant Activity and Nutritional Properties of Durum Wheat Grain

Cereals are a good source of antioxidant compounds, which include polyphenols in particular [97], and also raffinose, *N*-compounds, free aromatic amino acids, and pigments [24,61,62,90]. The levels of the carotenoids were not affected by the heat stress in the present study, which is in agreement with the strong Genotype component and the low Genotype × Environment interaction of this trait [98]. Otherwise, the higher carotenoid content observed for “Primadur” with respect to “T1303” is in agreement with studies that have indicated that carotenoid levels are higher in modern durum wheat varieties compared to older durum wheat varieties, and wild populations and landraces; this is due to the breeding activities where an increasing yellow color became important over the last few decades [23]. Similar data have been reported for other species, such as peach, where there is evidence that the carotenoid content is higher in yellow flesh than in white- and red-flesh peach genotypes [99].

Anthocyanins derive from the metabolism of aromatic amino acids, and for “T1303”, the anthocyanin levels increased significantly in response to the heat stress, which is in agreement with a previous study carried out in wheat [69]. In mature seeds under control conditions, the levels of aromatic amino acids were higher for “T1303” (purple grain) than for “Primadur” (yellow grain), which is in agreement with data from red and purple potato genotypes with respect to white and yellow ones [92]. Furthermore, the levels of aromatic amino acids and a product of their metabolism, ferulic acid, were higher for the mature grain of “Primadur” from the heat-stressed plants compared to the mature grain of “Primadur” plants grown under control conditions, with the opposite effect for “T1303”, where the heat stress induced decreases in the levels of these compounds.

The significant increase in the anthocyanins induced by heat stress for the mature seeds of “T1303” can explain these differences, as aromatic amino acids are used in the biosynthetic pathway of the anthocyanins. A similar hypothesis can be proposed in relation to the different accumulation of the sugars (which include raffinose), which were more abundant for the “T1303” mature seeds from the control plants, and lower for the “T1303” mature seeds from the heat-stressed plants, compared to “Primadur”. Similar data were reported in a previous study in potato in which Payyavula *et al.* [92] suggested that the regulatory loop of expression of the anthocyanin 1 transcription factor gene *AN1* can liberate hexoses that are used in the phenylpropanoid pathway.

For the antioxidant activity, it is known that no single method is adequate to evaluate this in complex samples, as many individual compounds are known to contribute to the antioxidant capacity of foods or biological samples [61,100]. The Trolox equivalent antioxidant capacity (TEAC) method which was used to evaluate antioxidant capacity in the present study considers most of the compounds with antioxidant properties analyzed in the present study, including phenolic compounds [34], free aromatic amino acids (*i.e.*, Tyr, Trp), polyamines, β-carotene metabolites, and the different β-carotene isomers [24,61,101,102]. This method does not measure the antioxidant capacity of cyanidin 3-glucoside, the most abundant anthocyanin metabolite in wheat, but instead it includes the phenolic compounds that are highly correlated to the kernel pigmentation [103,104,105]. Therefore, the increase in the antioxidant activity seen for the “T1303” seeds derived from the heat-stressed plants is probably due to the activity of the bulk polyphenolic compounds, which were not measured in the present study, as these correlate to the increase in the kernel pigmentation (*i.e.*, the anthocyanin content). For “Primadur”, the antioxidant activity will be mainly related to the carotenoid pigment content (or the related phenolic compounds), the levels of which did not change here following the heat stress. At the same time, the increased levels of polyamines, ferulic acid, and aromatic amino acids observed for “Primadur” did not increase the antioxidant capacity of this grain.

Although an increase in the antioxidant capacity for “Primadur” in response to the heat stress was not seen in the present study, the accumulation of polyamines in response to the heat stress can have beneficial effects on human health. The major roles of polyamines in the prevention of chronic diseases [62,106] and in the regulation of inflammatory reactions and differentiation of immune cells [107] are well known. In plants, elevated levels of polyamines are one of the most remarkable changes that occur in response to abiotic stress conditions [70,108]; however, in response to heat stress, there was only an increase for “Primadur”. Raffinose also increased in response to the heat stress for “Primadur”, and it is known to have antioxidant and anti-nutritional properties because it cannot be digested by monogastric animals [109,110]. Therefore, our data indicate that heat stress can affect the accumulation of these compounds in different ways, which can result in either beneficial or detrimental effects on human health in relation to the wheat genotype and the environmental conditions that occur during the plant growth.

## 4. Materials and Methods

### 4.1. Plant Material and Heat-Stress Treatment

Two durum wheat genotypes were chosen for this study: “Primadur” (Blondur//2587-8-6-/Leeds) [111], an élite cultivar with high grain yield and carotenoid content [23,112], and an Ethiopian purple durum wheat genotype, “T1303” (USDA code PI 352395) with high levels of anthocyanins in the grain [34].

Heat stress was applied following the protocol previously described by Laino *et al.* [13]. Briefly, the two genotypes were grown up to the third leaf stage in a climate chamber in a medium of soil, sand, and peat (6:3:1) at 10 °C (9 h day)/7 °C (15 h night), with 60% relative humidity and a photon flux of 500 µmol·m^−2^·s^−1^. At this point, the conditions were gradually changed (according to the developmental stage) to 20 °C (13 h day)/17 °C (11 h night), with 55% relative humidity. The heat stress was applied at five days after flowering. While the conditions for the control plants remained unchanged, the heat-stressed plants were transferred to a different growth chamber at 37 °C (13 h day)/17 °C (11 h night), with the same relative humidity for five days.

Following the heat shock, the temperature was decreased to 28 °C for 4 h, and then the growing cycle was changed to the same conditions before stress. The first sampling was carried out four days after the heat-stress treatment (*i.e.*, immature seeds, at 14 days after flowering) for the metabolic profiling. From the milk to maturity stage, both control and stressed plants were maintained at 25 °C (16 h day)/20 °C (8 h night), with 45% relative humidity. Physiologically mature seeds were also collected. The analyses were carried out for all of the parameters on the seeds from the principal spike, and were sampled from three biological replicates. The samples used to analyze the qualitative/nutritional parameters were stored at 4 °C before processing. The samples used for the metabolic profiling were immediately frozen in liquid nitrogen and freeze-dried, and stored at −80 °C until analysis. These analyses were conducted on the ground samples. The analyses were all conducted in triplicate, and the data are expressed on a dry weight (dw) basis.

### 4.2. Yield-Related and Qualitative/Nutritional Traits

#### 4.2.1. Yield-Related Traits

Six principal spikes were harvested from three biological replicates, and the individual grain weights and grain yields per spike were measured, at both 14 DAF and at physiological maturity. The individual grain weight was measured in triplicate for each of the three biological replicates, as the mean of 50 grains collected from two spikes and expressed in milligrams. The grain yield per spike was measured for each biological replicate as the mean for two spikes and expressed in milligrams. These data provided an indication of the difference between the two genotypes and the efficiency of the heat-stress treatment.

#### 4.2.2. Protein Content

The total nitrogen content was determined using the micro-Kjeldhal method [113] (AACC method 46-13.01). The grain-protein percentage was calculated by multiplying the Kjeldhal nitrogen by the conversion factor of 5.7, with this expressed on a dw basis.

#### 4.2.3. Total Anthocyanin Content Using the pH Differential Method

The extraction and purification of anthocyanins was performed according to the method of Hosseinian [69], with some modifications [34]. A mixture of methanol acidified with 1 N HCl (85:15; *v*/*v*) (8 mL) was added to the wholemeal sample (0.5 g), and then sonicated for 18 min at room temperature in an ultrasonic bath. After centrifugation at 9000× *g* for 15 min at room temperature, the supernatant was recovered. The pellet was extracted with 4 mL acidified methanol, and subjected to further centrifugation. The supernatants were then pooled and incubated at −20 °C in the dark for 48 h to facilitate macromolecule precipitation. The sample was then centrifuged at 9000× *g* for 15 min, and the supernatant was filtered using 0.45 µm regenerated cellulose syringe filters. Total anthocyanin content was evaluated using a colorimetric method at different pHs. Two dilutions of the samples were prepared, one for pH 1.0 using potassium chloride buffer (0.03 M KCl), and the other for pH 4.5 using sodium acetate buffer (0.4 M CH_3_CO_2_Na·3H_2_O). These samples were incubated for 30 min at room temperature in the dark and then filtered with 0.45 µm regenerated cellulose syringe filters. The absorbance of each sample was measured at 520 nm and the concentration of anthocyanin was calculated according to Hosseinian *et al.* [69]. The total anthocyanin content was corrected for dw and is expressed as cyanidin-3-glucoside equivalents in µg/g dw.

#### 4.2.4. Total Carotenoid Content

The total carotenoid content was analyzed according to method 14-50 of AACC International [114], with some modifications [115].

#### 4.2.5. *In-Vitro* Determination of Antioxidant Activity

The antioxidant activity was evaluated using the TEAC, or ABTS-2,2′-azinobis-(3-ethylbenzothiazoline-6-sulfonic acid), method. This method is based on the reduction of the blue-green cation radical ABTS^+^ at 734 nm due to antioxidants present in the sample tested. The decrease in absorbance is expressed as percentage inhibition of ABTS^+^, and is determined as a function of the concentration and the time, which is then calibrated against Trolox as the reference standard [116]. These data are expressed as mmol Trolox per kg dw.

### 4.3. Analysis of Polar Metabolites

Seeds at the physiological maturity stage were freeze-dried to determine the dry weight, and then milled using a mill (Pulverisette^®^ 7 Planetary Micro Mill; Classic Line, Fritsch GmbH Milling and Sizing, Idar-Oberstein, Germany) with an agate jar and balls, and then stored at −80 °C until analysis. Immature seeds were ground to a fine powder in a mortar with liquid nitrogen, freeze-dried and then stored at −80 °C until analysis. The extraction, derivatization and analysis of these samples for the profiling of the polar metabolites were performed by gas chromatography linked to mass spectrometry (GC-MS), following protocols described previously [117]. All of the analyses were performed in three technical replicates for each of three biological replicates. Briefly, 100 mg dw of each sample were extracted using a mixture of methanol (1 mL), ultrapure water (1 mL), and trichloromethane (3 mL), added sequentially. The samples were stored at 4 °C for 30 min, and then centrifuged at 4000× *g* for 10 min at 4 °C. Aliquots (50 µL) of the polar phase were dried in a Speedvac, for further analysis. The polar fraction was redissolved and derivatized for 90 min at 37 °C in methoxyamine hydrochloridein pyridine (70 µL; 20 mg/mL), followed by incubation with *N*-methyl-*N*-(trimethylsilyl) trifluoroacetamide (120 µL) at 37 °C for 30 min. The polar metabolites were analyzed using GC (Agilent 6890N; Agilent Technologies, Santa Clara, CA, USA) coupled with quadruple MS (Agilent 5973, Agilent Technologies). Samples (1 µL) were injected in the splitless mode, with GC separation on an HP-5ms capillary column (60 m, 0.25 mm i.d., 0.25 mm film thickness). Helium was used as the carrier gas, at a constant flow rate of 1 mL/min. For the analysis of polar metabolites, the injection temperature, transfer line, and ion source were set at 280 °C, and the quadrupole was adjusted to 180 °C. The oven was kept at 70 °C for 1 min, then increased at a rate of 5 °C/min to 310 °C, and held for 15 min. Subsequently, the temperature was increased to 340 °C and held for 1 min. The spectrometer was operated in electron-impact mode at 70 eV, the scan range was from 30 to 700 amu (atomic mass units), and the mass spectra were recorded at 2.21 scan/s. The standards and all of the chemicals used were from Sigma Aldrich Chemical Co. (HPLC grade; Deisenhofen, Germany), and *N*-methyl-*N*-(trimethylsilyl) trifluoroacetamide was from Fluka (Deisenhofen, Germany).

The polar metabolites were identified by comparing the MS data with those of the National Institute of Standards and Technology 2008 database, and with a custom library compiled with reference compounds. The GC-MS quantification was performed using the Chemstation software. The absolute concentrations of the polar metabolites were determined by comparisons with standard calibration curves obtained in the range of 0.04 to 2.00 ng before the GC-MS injections and expressed as µg/g dw. The samples were randomized, with the instrumental performance monitored by internal standards (Ribitol, 20 µL; 0.2 mg/mL) added after the extractions.

### 4.4. Statistical Analysis

The analysis of variance (ANOVA) was carried out with respect to each analytical compound detected in whole-grain material during grain filling and at maturity stage; the effect of genotypes was assessed according to a completely randomized design (with three repetitions). A standard procedure for the analysis of variance was applied to the data, to separate the effects due to Genotype (G), Treatment (T), timing (t), and their interactions. Statistically significant differences were determined at the probability level of *p* < 0.05. The magnitude of each factor was determined by components of variance analysis. Due to the differences in seed weight which were observed between the two genotypes, and inside each genotype following the heat stress, ANCOVA analysis was also carried out to correct the data with respect to seed weight. As the results were consistent with those of ANOVA, only ANOVA results are shown in the present study. All statistical analyses were performed using the STATISTICA program (StatSoft, vers. 7.1, 2005, Agilent Technologies, Santa Clara, CA, USA).

## 5. Conclusions

Twelve different mega-environments have been described for wheat cultivation worldwide based on cropping systems (e.g., rain-fed *versus* irrigated, spring *versus* winter) and the presence of biotic and abiotic stress [3,118]. Most of these constraints are expected to increase in the framework of climate change. Therefore, new genotypes that are well adapted to these environments have to be selected, particularly when considering the trends towards increasing temperatures. Improved genotypes have to be selected not only in terms of their productive capacity, but also on the basis of their qualitative and nutritional parameters, which are strongly influenced by heat stress. The present study shows that heat stress applied to wheat plants early during seed development can have strong effects on the mature wheat grain in terms of the metabolite accumulation and the nutritional and antioxidant properties, and that these responses are strictly dependent upon the genotype. Also, even if the genetic background of two durum wheat genotypes is different, it cannot be excluded that some of the differences observed in the responses to heat stress will be due to anthocyanin accumulation, which can be considered a trait of interest for future breeding activities with wheat. Further studies are needed to better clarify the effect of anthocyanin accumulation on the response to heat stress. The analysis of a large number of yellow and red-grain genotypes and the development of near-isogenic lines, with the background of a yellow-grain cultivar and the locus/loci for anthocyanin synthesis, may be suitable approaches to face this aspect.

## Supplementary Materials

Supplementary materials can be found at http://www.mdpi.com/1422-0067/16/12/26241/s1.
