# Supplementary material for: Effects of Heat Stress on Metabolite Accumulation and Composition, and Nutritional Properties of Durum Wheat Grain
Source: Int J Mol Sci. 2015 Dec 19;16(12):30382–404. doi: 10.3390/ijms161226241 (PMC4691181; doi:10.3390/ijms161226241)
Supplement: Supplementary file 1 [file ijms-16-26241-s001.pdf]

Supplementary Material

Effects of Heat Stress on Metabolite Accumulation and Composition, and Nutritional Properties of Durum Wheat Grain

Anna Maria De Leonardis, Mariagiovanna Fragasso, Romina Beleggia, Donatella Bianca Maria Ficco, Pasquale De Vita and Anna Maria Mastrangelo

Table S1. ANOVA analysis of Genotype, timing and Treatment for each class of metabolite.

Variance Component		Amino Acids	*N*-Compounds	Organic Acids	Sugars	Sugar Alcohols
Genotype	"Primadur"	6189 [a]	48.01 [a]	8738 [a]	228,679 [a]	1226 [a]
	"T1303"	3895 [b]	6.86 [b]	3668 [b]	142,187 [b]	745 [b]
	Significance	***	***	***	***	***
timing	Immature	7757 [a]	43.72 [a]	8075 [a]	260,274 [a]	1745 [a]
	Mature	2327 [b]	11.15 [b]	4331 [b]	110,592 [b]	226 [b]
	Significance	***	***	***	***	***
Treatment	Control	4551 [b]	22.34 [b]	5807 [b]	177,697 [b]	952 [b]
	Heat-shocked	5534 [a]	32.54 [a]	6599 [a]	193,169 [a]	1019 [a]
	Significance	***	**	**	*	*

*, p <0.05; **, p <0.01; ***, p <0.001; Values in the same column followed by different letters are significantly different ($p < 0.05$).

Table S2. ANOVA analysis of Genotype × Treatment interactions for each class of metabolite.

Genotype × Treatment		Amino Acids	*N*-Compounds	Organic Acids	Sugars	Sugar Alcohols
"Primadur"	Control	4424 [b]	34.5 [b]	5945 [b]	177,865 [b]	1098 [b]
	Heat shocked	7955 [a]	61.5 [a]	11,531 [a]	279,494 [a]	1355 [a]
"T1303"	Control	4677 [b]	10.1 [c]	5668 [b]	177,529 [b]	807 [c]
	Heat shocked	3113 [c]	3.5 [c]	1668 [c]	106,845 [c]	684 [d]
	**Significance**	***	***	***	***	***

***, $p < 0.001$; Values in the same column followed by different letters are significantly different ($p < 0.05$).

Table S3. Significance levels in the comparisons of the differences in the levels of the metabolites under control conditions with respect to heat-shocked conditions (Student's *t* tests).

Metabolite Class	Metabolite	Control *versus* Heat Shocked Significance Level According to Seeds and Genotype			
		Immature Seeds		Mature Seeds	
		"Primadur"	"T1303"	"Primadur"	"T1303"
Amino acids	Glutamic acid	0.12	0.030	0.002	0.0012
	β-Alanine	0.2	0.02	0.0003	0.001
	Asparagine	0.02	0.01	0.008	0.0008
	Aspartic acid	0.001	0.005	0.0003	0.0006
	GABA	0.013	0.004	0.001	0.0001
	Threonine	0.40	0.33	0.0001	0.00006
	Serine	0.19	0.16	0.001	0.018
	Glycine	0.02	0.052	0.013	0.0054
	Isoleucine	0.07	0.08	0.016	0.0026
	Leucine	0.27	0.28	0.001	0.042
	Valine	0.80	0.10	0.001	0.056
	Alanine	0.58	0.0001	0.001	0.016
	Phenylalanine	0.001	0.078	0.000004	0.008
	Tyrosine	0.04	0.20	0.004	0.02
	Tryptophan	0.04	0.062	0.009	0.017
N-compounds	Putrescine	0.025	0.67	0.024	0.48
	Cadaverine	0.006	0.11	0.001	0.071
	Spermidine	0.39	0.44	0.011	0.0004
Organic acids	Oxalic acid	0.001	0.0002	0.023	0.048
	Fumaric acid	0.001	0.0003	0.0001	0.0001
	Ferulic acid	0.00003	0.104	0.0003	0.0004
	Quinic acid	0.97	0.002	0.008	0.0011
	Citric acid	0.016	0.001	0.017	0.002
	Shikimic acid	0.53	0.051	0.08	0.37
	Malic acid	0.002	0.014	0.002	0.0006
	Saccaric acid	0.042	0.003	0.0004	0.001
	Nicotinic acid	0.310	0.236	0.0236	0.011
	3PGA	0.001	0.500	0.0003	0.0008
Sugars	Raffinose	0.245	0.002	0.014	0.028
	Sucrose	0.012	0.010	0.0096	0.0044
	Glucose	0.0001	0.104	0.0014	0.009
	Glucose 6-phosphate	0.305	0.075	0.014	0.037
	Fructose	0.002	0.191	0.0054	0.023
	Fructose 6-phosphate	0.128	0.114	0.024	0.017
	Ribose	0.005	0.134	0.0024	0.0089
	Mannose	0.008	0.80	0.0001	0.0066
	Palatinose and maltitol	0.001	0.028	0.42	0.0037
	Xylose and lixilose	0.00003	0.045	0.0003	0.016
	Maltose and turanose	0.003	0.250	0.0003	0.00005
Sugar alcohols	Mannitol	0.42	0.0002	0.0007	0.0009
	myo-Inositol	0.026	0.009	0.0005	0.0005
	Glycerol	0.704	0.451	0.026	0.0084

GABA, γ-4-aminobutyrric acids; 3PGA, 3-phosphoglyceric acid.
